# Primary Categorizing and Masking Cerebral Small Vessel Disease Based on “Deep Learning System”

**DOI:** 10.3389/fninf.2020.00017

**Published:** 2020-05-25

**Authors:** Yunyun Duan, Wei Shan, Liying Liu, Qun Wang, Zhenzhou Wu, Pan Liu, Jiahao Ji, Yaou Liu, Kunlun He, Yongjun Wang

**Affiliations:** ^1^Department of Radiology, Beijing Tiantan Hospital, Capital Medical University, Beijing, China; ^2^National Center for Clinical Medicine of Neurological Diseases, Beijing, China; ^3^Department of Neurology, Beijing Tiantan Hospital, Capital Medical University, Beijing, China; ^4^Beijing Institute for Brain Disorders, Beijing, China; ^5^Laboratory of Translational Medicine, Chinese PLA General Hospital, Beijing, China; ^6^Key Laboratory of Ministry of Industry and Information Technology of Biomedical Engineering and Translational Medicine, Chinese PLA General Hospital, Beijing, China

**Keywords:** cerebral small vessel diseases (CSVD), deep learning system (DLS), categorizing, subcortical infarction, white matter hyperintensity, launce, cerebral microbleed, diagnosis-assistance

## Abstract

**Objective:**

To supply the attending doctor’s diagnosis of the persisting of cerebral small vessel disease and speed up their work effectively, we developed a “deep learning system (DLS)” for cerebral small vessel disease predication. The reliability and the disease area segmentation accuracy, of the proposed DLS, was also investigated.

**Methods:**

A deep learning model based on the convolutional neural network was designed and trained on 1,010 DWI b1000 images from 1010 patients diagnosed with segmentation of subcortical infarction, 359 T2^∗^ images from 359 patients diagnosed with segmentation of cerebral microbleed, as well as 824 T1-weighted and T2-FLAIR images from 824 patients diagnosed with segmentation of lacune and WMH. Dicw accuracy, recall, and f1-score were calculated to evaluate the proposed deep learning model. Finally, we also compared the DLS prediction capability with that of 6 doctors with 3 to 18 years’ clinical experience (8 ± 6 years).

**Results:**

The results support that an appropriately trained DLS can achieve a high-level dice accuracy, 0.598 in the training section over all these four classifications on 30 patients (0.576 for young neuroradiologists), validation accuracy is 0.496 in lacune, 0.666 in WMH, 0.728 in subcortical infarction, and 0.503 in cerebral microbleeds. It is comparable to attending doctor with a few years of experience, regardless of whether the emphasis is placed on the segmentation or detection of lesions with less time-spending compared with manual analysis, about 4.4 s/case, which is dramatically less than doctors about 634 s/case.

**Conclusion:**

The results of our comparison lend support to the case that an appropriately trained DLS can be trusted to the same extent as one would trust an attending doctor with a few years of experience, regardless of whether the emphasis is placed on the segmentation or detection of lesions.

## Introduction

The Cerebral Small Vessel Disease (CSVD) is an umbrella term covering a variety of abnormalities related to small blood vessels in the brain, which can be caused by many diseases, such as plaque accumulation in the small vessel, small vessel inflammation, and persisted chronic damage in the small vessel (hypertension) (Go et al., 2012; Rincon and Wright, 2014). Consequently, it could lead to irreversible consequences such as stroke, dementia, mood disturbance, and gait problems. The CSVD can be diagnosed by medical professionals based on magnetic resonance imaging (MRI) (Noguchi et al., 1997; Greenberg et al., 2009; Debette and Markus, 2010). Signs of CSVD on conventional MRI include lacunes, white matter hyperintensities (WMH), recent small subcortical infarcts, prominent perivascular spaces, cerebral microbleeds, and atrophy (Wardlaw et al., 2013). CSVD has been suggested to be an essential source of morbidity associated with ischaemic and hemorrhagic stroke, dementia, and depression (Pantoni, 2010). So it is critical to define the severity of CSVD by a quantitative assessment from MRI, which is relevant to the risk of stroke. However, the severity of CSVD is mainly evaluated by manual semi-quantitative or qualitative methods at present, which is time-consuming, laborious, and subjective (Rensma et al., 2018).

Nowadays, the deep convolutional neural networks (CNN) has proven to be useful and effective in medical applications, such as the classification (Mohsen et al., 2018) and segmentation (Havaei et al., 2017) of brain tumor problem as well as various vessel diseases (Dou et al., 2016; Ghafoorian et al., 2017). Besides, computers are immune to fatigue or emotions and can function 24 h daily. Moreover, a high-quality automatic segment can probably help doctors to speed up their diagnosis, and hence allowing more patients to be processed. In recent studies, deep learning has applied in stroke imaging data in areas including automated featurization, image segmentation, and multimodel prognostication (Huang et al., 2010; Misra et al., 2010; Kamnitsas et al., 2015; Stier et al., 2015). One of the significant strengths of deep learning is that there is no obvious solution that could be obtained manually, such as the prediction of poststroke MRI fluid-attenuated inversion recovery (FLAIR) changes given acute diffusion-weighted imaging‘(DWI) maps (Stier et al., 2015). Currently, the application of deep learning in CSVD is seldom reported. Several deep learning models for segmentation have been applied in three-dimensional images and worked well (Kamnitsas et al., 2017; Aslani et al., 2019). Nevertheless, two-dimensional data is commonly used in clinical practice.

To supply people with consistent and efficient CSVD area segmentation systems and help the young doctors to speed up their workflow, we developed a DLS for automatic area segmentation. Furthermore, to check the reliability of this system, we seek to investigate the relative performance between the proposed DLS system and human doctors on detecting and locating four types of CSVD (lacune, WMH, subcortical infarction and cerebral microbleeds) using T1-weighted, T2^∗^, T2-FLAIR, and DWI b1000 sequences. Prominent perivascular spaces and atrophy were not included in the DLS system, for they are difficult to make a reasonable manual evaluation in conventional two-dimensional images. Specifically, we compare the performance of the proposed DLS with the average performance of six doctors. Accurate lesion segmentation and identification can guarantee objective and accurate quantitative evaluation. The purpose of this study is to validate whether an appropriately-trained DLS can be trusted to the same extent as one would trust a doctor with a satisfying experience. Then the system may be applied to quantify the lesion load of CSVD and further to help establish the risk factor prediction model.

## Materials and Methods

### Standard Protocol Approvals, and Patient Consents

All the patients provided consent for access to the image data in this study. This study was approved by the ethics committee of the Beijing Tiantan Hospital and fulfilled the Helsinki Declaration.

### Data Quality Control

For the image quality evaluation, the three-point scale was applied: 1, “poor” (limited image quality that affects diagnosis); 2, “good” (minor artifacts or mildly reduced signal-noise ratio with no effects on diagnosis); and 3, “excellent” (no artifacts and optimal). Only scale 2 or 3 were allowed to be included in this study. More details of manufacturer and resolution information in Supplementary Figure 1.

### Image Dataset

We obtain 1500 anonymized patients data from Beijing Tiantan hospital and other 12 hospitals across China which are included in The Third China National Stroke Registry (CNSR-III). The MRI data include T1 weighted image (T1WI), T2^∗^, T2-FLAIR, DWI b1000 and TOF-MRA., The inclusion criteria of patients were: (Rincon and Wright, 2014) Age older than 18 (Go et al., 2012) Ischemic stroke or Transient ischemic attack (TIA) (Greenberg et al., 2009) Informed consent from patient or legally authorized representative (Primarily spouse, parents, adult Children, otherwise indicated) (Debette and Markus, 2010) The presence of one CSVD sign or more on MRI (Kamnitsas et al., 2015). Patients who had other abnormalities such as hemorrhage or brain tumor on MRI and well-defined macro-vascular stenosis on MRA were excluded.

**FIGURE 1:**
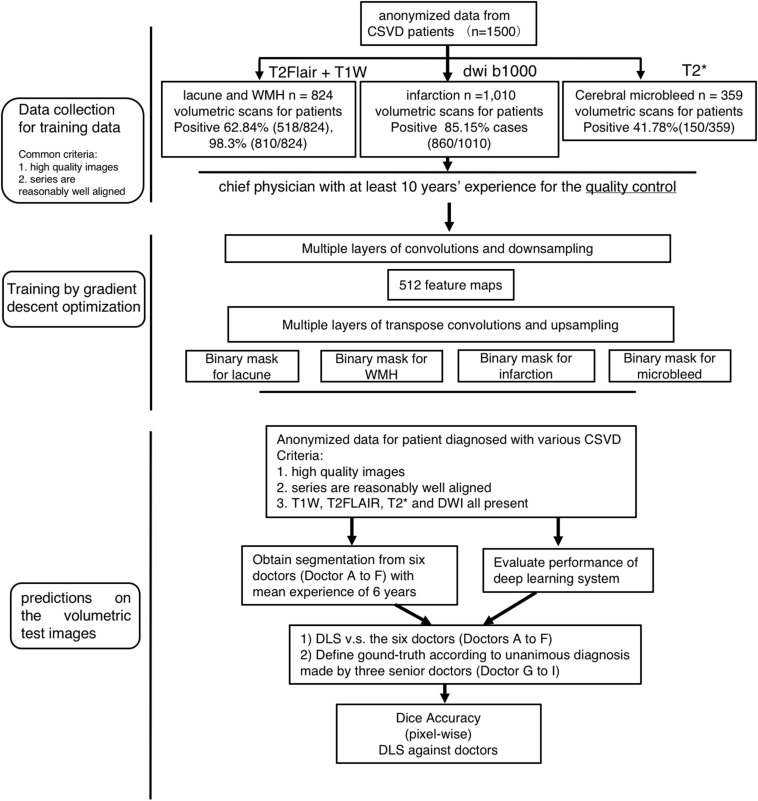
Flowchart of the patients’ distribution in training and clinical evaluation. The distribution and classification of all samples in each step was used for the model training, and clinical evaluation steps.

In total, we have 824 T1-weighted and T2-FLAIR images from 824 patients with segmentation of lacune and WMH, 1,010 DWI b1000 images from 1010 patients with segmentation of subcortical infarction, as well as 359 T2^∗^ images from 359 patients with segmentation of cerebral microbleed. Each volumetric MRI has a vertical spacing of between 6 and 8 mm. For each image, the spacing along the x- and y-direction varies from 0.36 to 1.44 mm between consecutive pixels. The distribution of pixel spacings for each dataset are shown in Figure 1. Instead of resizing the images to ensure a uniform pixel spacing, we train the model to be scale-invariant within the reasonable range of resolutions encountered in MRI.

**FIGURE 2:**
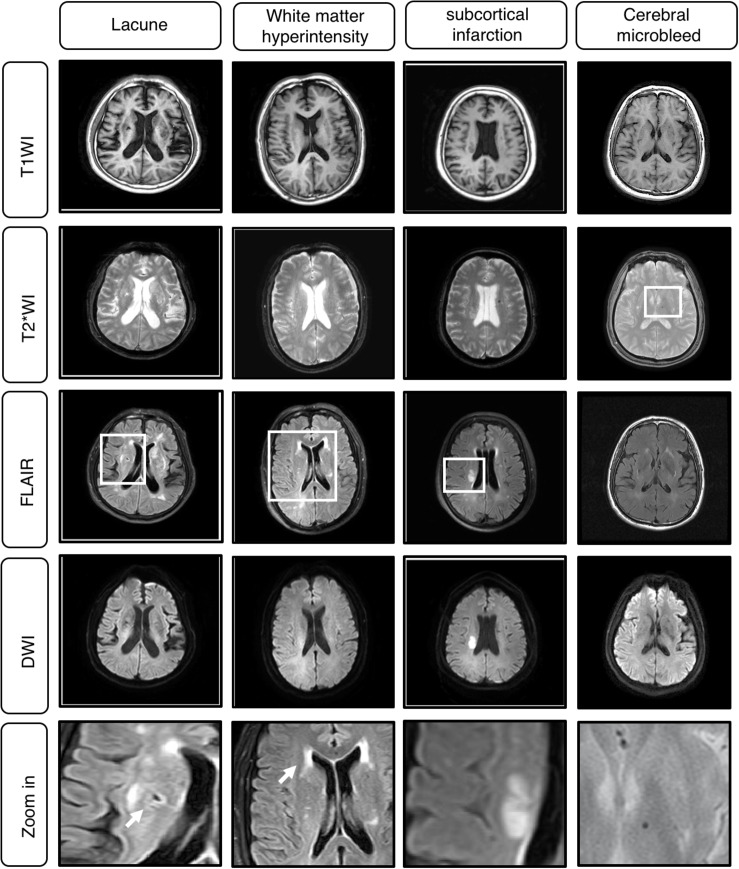
Example cases of Cerebral Small Vessel Disease (CSVD) MRI A. Classical MRI of CSVD including lacune, white matter hyperintensity (WMH), subcortical infarction, cerebral microbleed.

The segmentation labels for patients with lacune, WMH, subcortical infarction, or cerebral microbleed are endorsed by two radiologists with 12 years of clinical experience. The lacunes were mainly labeled on T1WI (CSF-like hypointensity) with referred to T2-FLAIR. The segmentation labels with WMH were based on T2-FLAIR. The subcortical infarction was labeled on DWI b1000 images. The segmentation labels with cerebral microbleed were labeled on T2^∗^-weighted GRE, with other sequences as reference. All the segmentation character of MRI illustrated in Tables 1, 2 and Figure 2.

**TABLE 1:** The definitions of imaging characteristics for CSVD on MRI.

	Lacunes	White matter hyperintensity	subcortical infarct	Cerebral microbleed
DWI	↓/↔	↔	↑	↔
T1	↓CSF-like	↓/↔	↓	↔
T2-FLAIR	↓/↔	↑	↑	↔
T2^∗^-weighted GRE	↓/↔ if haemorrhage	↔	↔	↔
Diameter	3 to 15 mm	Variable	≤20 mm	2 to 10 mm

*↑ signal increased, ↓ signal decreased, ↔ equal signal.*

**TABLE 2:** Clinical symptom distribution in the evaluation dataset (*n* = 30).

	Lacune	White matter hyperintensity	subcortical infarct	Cerebral microbleed
Positive symptom	30	27	29	30
Negative symptom	0	3	1	0

### Evaluation Dataset and Reference Standard

The evaluation dataset comprises 30 patients, with T1-weighted, T2^∗^, T2-FLAIR, and DWI b1000 sequences available for each patient. All these patients’ clinical diagnosis must meet the inclusion criteria and each patent’s image must have 2 to 4 signs of CSVD, and all of these patients are independed from the previous dataset.

We define the ground truth location of these four possible diseases according to the diagnosis and segmentation label by three senior physicians, with all giving their consensus. These three physicians who set the reference standard on the 30 patients are top expects on radiology in our hospital with 12, 13, and 15 years of experience, respectively.

### Credentials of Doctors Performing Segmentation on the Evaluation Dataset

After training on 1,500 patients MRI obtained from hospitals, we make predictions on 30 patients chosen by a hospital doctor randomly among patients who had T1-weighted, T2^∗^, DWI and FLAIR sequences in their records. The reference standard is prescribed unanimously by three senior doctors as described previous.

The six doctors in the evaluation test independently performing segmentation on the evaluation dataset include three resident physicians, each with three years of experience, an attending physician with nine years of experience, and two chief physicians with 14 and 18 years of experience, respectively. All the doctors included in the tests are neuroradiologists.

To ensure that the doctors are evaluated in their best state, they are requested to perform the segmentation to the best of their abilities, without any constraint on time or duration.

### Setting up of the Deep Learning Algorithm

The proposed DLS system which consists of four segementor subsystems was trained to learn features from MRI, extracted from four different types of CSVD diseases. To increase the rate of convergence of the network during training, preprocessing was done on each MRI to standardize them across various acquisition parameters. The histogram peaks were normalized and aligned based on the white matter content in the MRI.

**FIGURE 3:**
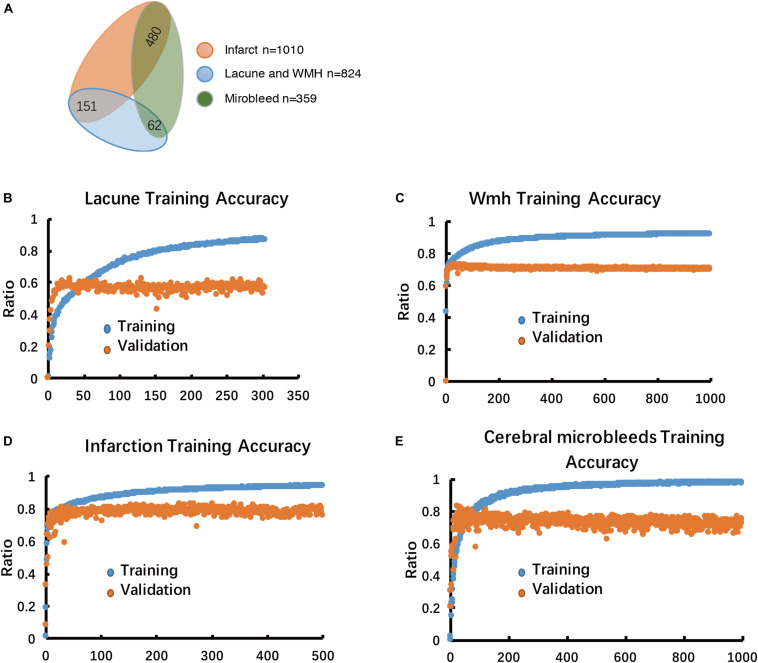
Multiple-label classification of CSVD, and Performance analysis of the model in the training stage. **(A)** Multiple-label classification of CSVD. **(B–E)** Model performance in training accuracy and validation accuracy.

The training set was consisted of 1500 patients with conventional MRI T1W, T2^∗^, T2-FLAIR, DWI b1000 image data, including Lacuna data (*n* = 824 volumetric scans, 98.3% positive cases) and WMH data (*n* = 824 volumetric scans, 98.3% positive cases), subcortical infarction data (*n* = 1010 volumetric scans, 85.15% positive cases), Cerebral microbleed data (*n* = 359 volumetric scans, 41.78% positive cases) (Figures 1, 3A). Each disease was trained independently by one segmentor subsystem of the DLS system. Each training was stopped when the training accuracy was greater than 98% and diverged from validation accuracy by more than 15%, as we think that at such time, the DLS has reached the optimal performance (Figure 3).

During the inference stage, patient’s MRI sequences are fed to the DLS system as input. Each segementor subsystem grabs its own sequences from the DLS system’s input and give a segmentation prediction of a certain disease. Before giving a final output of the DLS, the four segmentation predictions are combined in a way such that WMH, lacune and subcortical infarction are multually exclusive in the pixel level. Please note that it’s a multi-label classification problem in the image or patient level.

### Network Architecture

**FIGURE 4:**
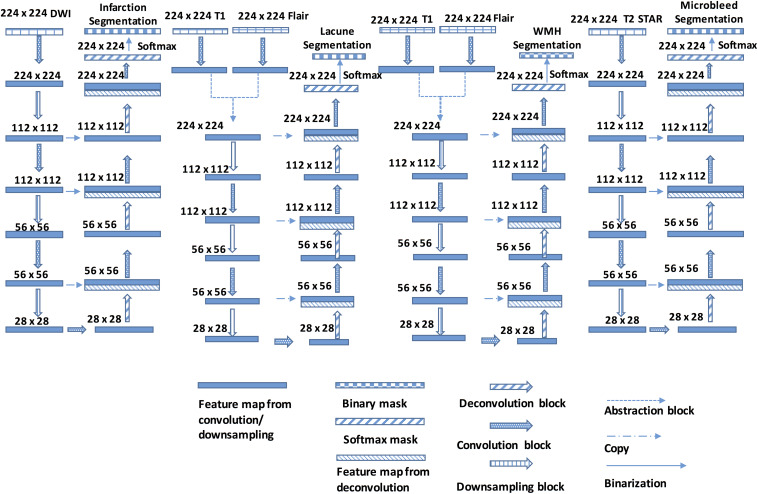
The Structure of the DLS used for CSVD detection and segmentation. Each Encoder block contains one or more convolution steps followed by max-pooling for downsampling. Each time the feature maps are downsampled, the number of output channels is increased. Each Decoder block comprises one deconvolution (transpose convolution) operation that upsamples the size of the feature maps and correspondingly reduces the number of output channels.

The proposed, end-to-end, DLS was composed of four segmentor networks (Figure 4). The preprocessing steps consisted of padding to square, resizing, and normalizing. Each segmentor network takes one or more MRI sequences as input and outputs a binary segmentation mask on a corresponding sequence.

All four segmentor networks are based on the widely-used U-Net architecture (Ronneberger et al., 2015; Havaei et al., 2016), and each of them predicts the mask of the disease area of one of four CSVD introduced above. For example, segmentor network #1 is used to detect brain subcortical infarction. It takes DWI b1000 as input and outputs a segmentation mask of subcortical infarction area. Those four segmentor networks are trained and validated independently, which allows the network to be optimized for detecting the CSVD, and are combined to perform the disease area prediction. For each patient, the volumetric MRI are separated into two dimensional images and, after certain preprocessing steps, fed to the DLS to generate masks of diseases area. During the postprocessing steps, the generated WMH mask is subtracted by the generated subcortical infarction mask, because subcortical infarction has a similar signal property to WMH on T2-FLAIR. For the same reason, the generated WMH mask is also subtracted by the generated lacune mask. Then, the generated lacune mask is subtracted by the generated subcortical infarction mask. Finally, the two dimensional segmentation predictions from our model are concatenated to obtain a complete three dimensional segmentation predictions of the patient (More detailed information in Figure 4).

### Algorithm for Segmented Images

To evaluate the performance of proposed segmentation networks, the commonly used metric known as the dice score (accuracy) was used (Sudre et al., 2017). The dice score is computed for each patient, and the arithmetic mean is taken.

A Free Response Operating Characteristic (FROC) analysis can be obtained in this study (Bandos et al., 2009). Due to binary rather than probabilistic diagnoses from doctors, rendering the comparison between our model and the doctors irrelevant. Adapting the concept of treating each lesion equally, we do away with the probabilistic element of FROC and compare the F1 score, of our model’s predictions after thresholding (Goutte and Gaussier, 2005).

Moreover, Region-wise F1 score also applied in this study, it provides another avenue for us to answer the research question of how the predictions made by a deep learning model compares with that of human doctors (Goutte and Gaussier, 2005).

Another evaluation metric as a less demanding alternative to the dice score was applied in this study. We discretize the reference mask as well as predictions into square grids with spacing approximately equal to the square root of the image dimensions. Each patch, which may be viewed as bins mapped from a neighborhood of pixels, will be classified positive for the disease as long as at least one pixel in that patch is positive, or be classified negative otherwise. This is equivalent to performing a max-pooling followed by resizing back to the original number of pixels. In the limit where the patch is equal to the image size, the segmentation problem becomes converted to a multiple-label classification problem.

### Statistical Analysis

The SPSS Statistics 23.0 software package for Windows (IBM Corp., Armonk, NY) was performed for statistical analyses.

## Results

It can be observed that our model possibly releases a prediction more faithful to the reference standard, compared to that of the doctors taking part in the clinical evaluation, regardless of whether the emphasis is placed on the segmentation or the detection of lesions. Where detailed pixel-level segmentation of lesions is required, our model’s dice accuracy of 59.8% is over two percentage points better than the doctors’ dice accuracy of 57.6% (Table 3). If the focus is on detect the presence of lesions, our model provides an average F1 score of over 72.5%, more than three percentage points over the doctors’ 69.1% (Table 3).

**TABLE 3:** Dice accuracy at pixel-wise criteria and F1 score for four CSVDs.

	Our model	Doctors
Dice Accuracy (Pixel-wise)	0.598	0.576
Region F1 score	0.725	0.691

*Dice accuracy at pixel-wise criteria and F1 score for four CSVDs of 30 patients according to the predictions made by six doctors (Neuroradiologists) and by our model, concerning the reference standard. In the pixel-wise evaluation, the images and masks have a resolution of 224 × 224 pixels.*

**FIGURE 5:**
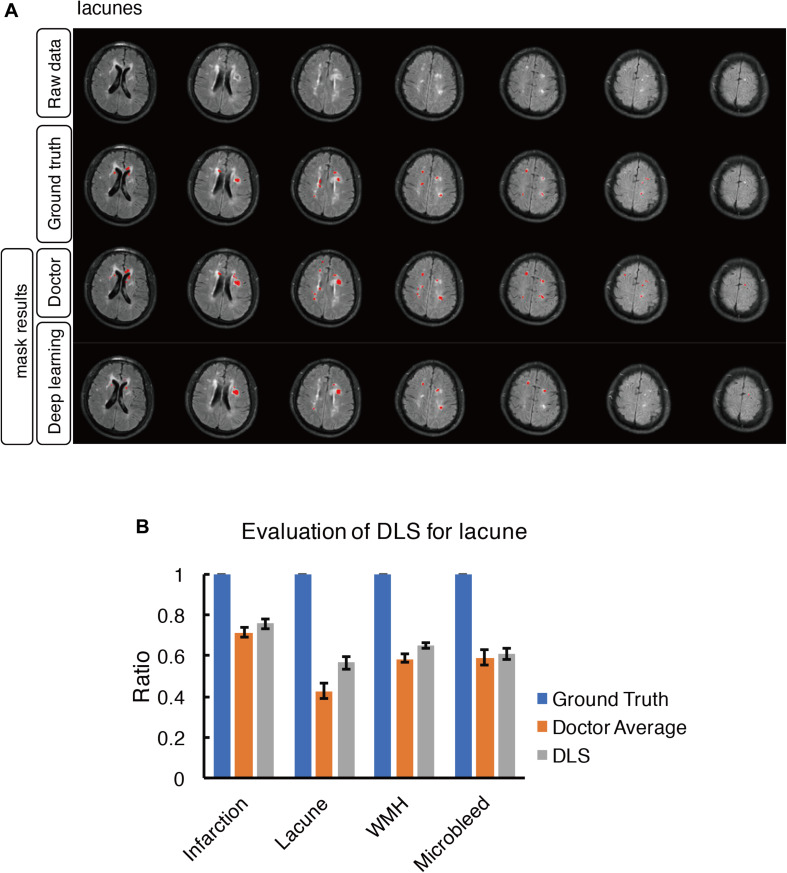
Mask result of lacune in the study, including Raw data, Ground truth, Doctor mask and DLS mask result. **(A)** showed multiple lacunes in the regions of bilateral paraventricular and semi-oval center, represented as well-defined CSF-like hypointensity on T1WI. The four rows are raw data, ground truth, doctor’s segmentation label and segmentation prediction from DLS, respectively. **(B)** showed the comparison of accuracy ratio of segmentation label from doctors with 95% confidence interval and DLS.

**FIGURE 6:**
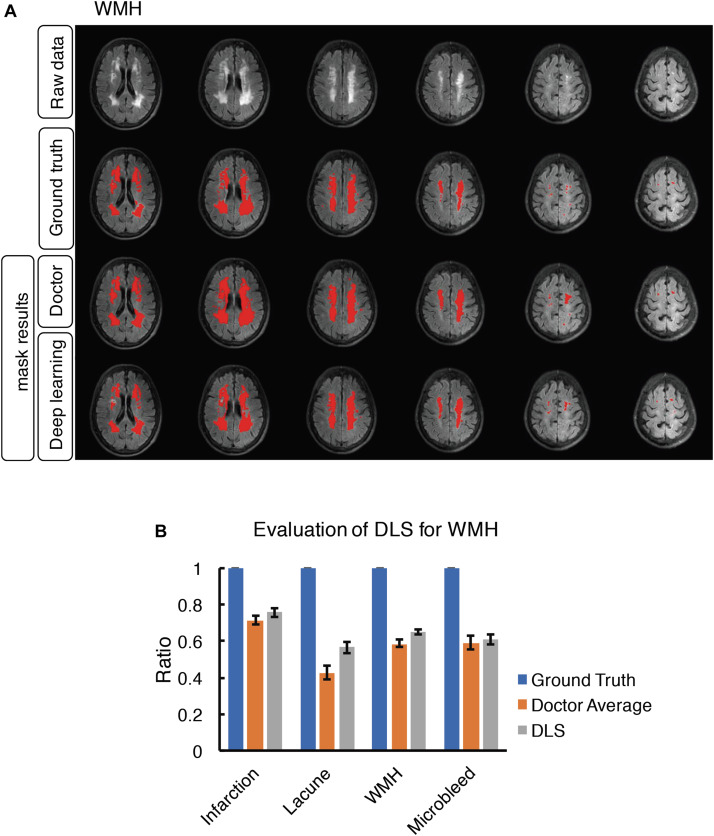
Mask result of WMH in the study, including Raw data, Ground truth, Doctor mask and DLS mask result. **(A)** showed hyperintensity in bilateral white matter regions of paraventricular and the frontal and parietal lobe on T2-FLAIR. The four rows are raw data, ground truth, doctor’s segmentation label and segmentation prediction from DLS. **(B)** showed the comparison of accuracy ratio of segmentation label from doctors with 95% confidence interval and DLS.

**FIGURE 7:**
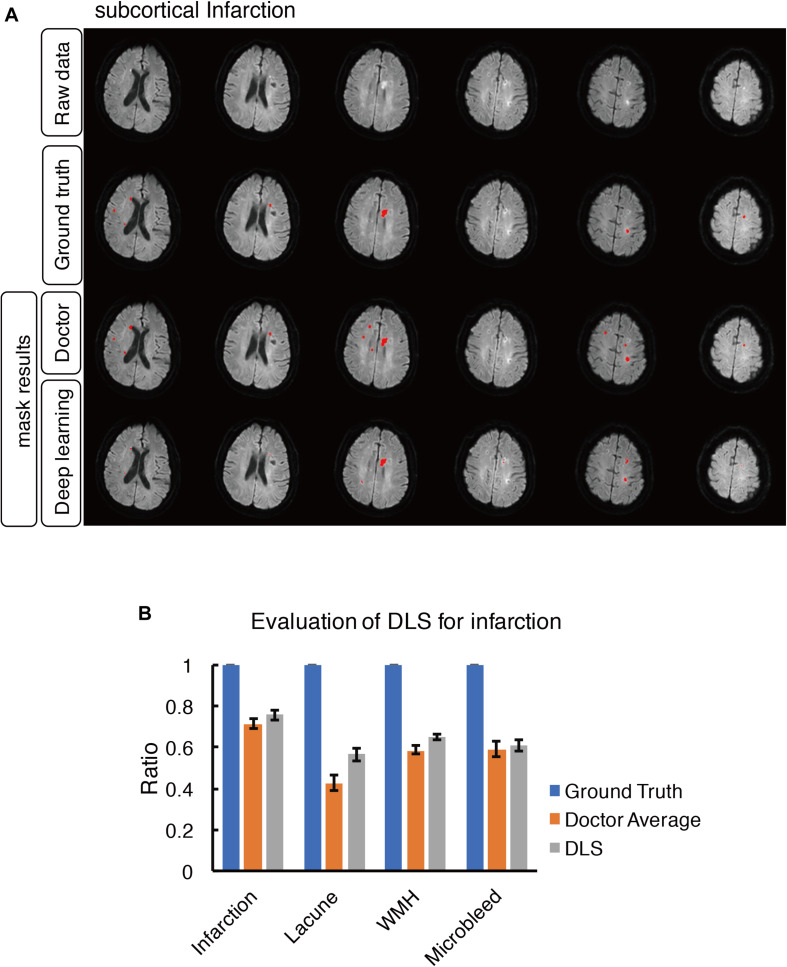
Mask result of subcortical infarction in the study, including Raw data, Ground truth, Doctor mask and DLS mask result. **(A)** The represented images showed multiple recent subcortical infarcts, represented as hyperintensity on DWI in regions of left corpus callosum, bilateral paraventricular and semi-oval center. The four rows are raw data, ground truth, doctor’s segmentation label and segmentation prediction from DLS, respectively. **(B)** Showed the comparison of accuracy ratio of segmentation label from doctors with 95% confidence interval and DLS.

**FIGURE 8:**
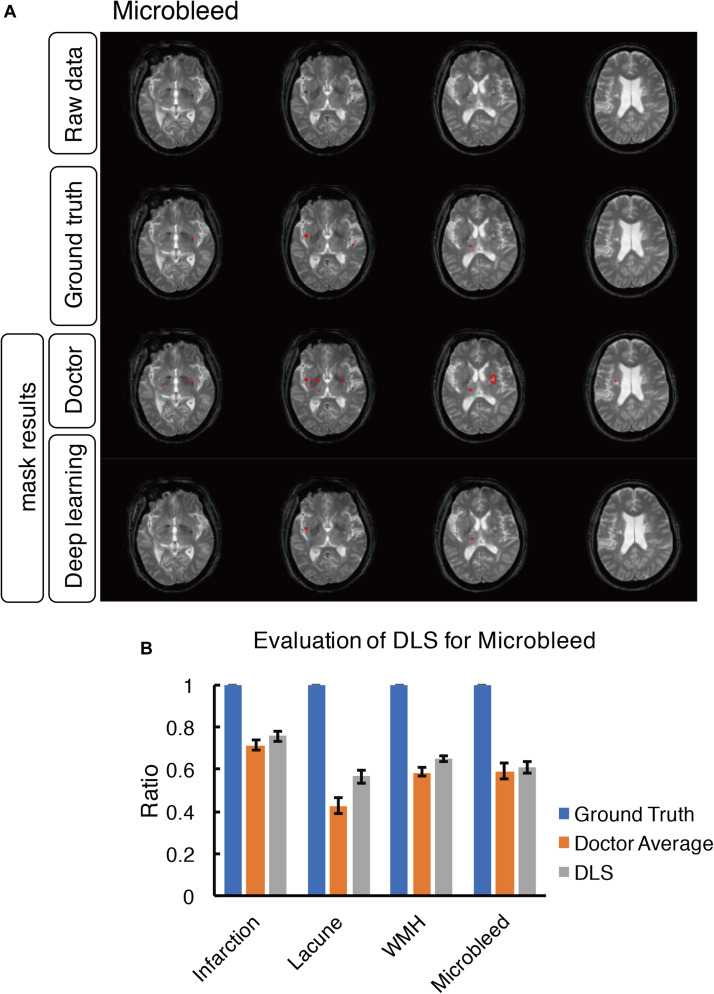
Mask result of cerebral microbleed in the study, including Raw data, Ground truth, Doctor mask and DLS mask result. **(A)** showed cerebral microbleed lesions in the right insula and right thalamus, represented as hypointensity on T2*WI. The four rows are raw data ground truth, doctor’s segmentation label and segmentation predation from DLS, respectively. **(B)** showed the comparison of accuracy ratio of segmentation label from doctors with 95% confidence interval and DLS.

Considering each of the four CSVD individually, the dice accuracy, as well as region-wise F1 score achieved by our model, is higher than that of the doctors in the segmentation of lacune, WMH and subcortical infarction, as can be verified from Table 4 and Figures 5–8. Given that our model, as well as the doctors, perform best on the segmentation of subcortical infarction. Depending on how we define success in terms of pixel-wise segmentation or the detection of lesions, and the tolerance for uncertainties of a few pixels, our model, attains a score with 0.728 in dice accuracy and 0.859 in region-wise F1 score, which is consistently similar to the doctors’ score with 0.714 in dice accuracy and 0.839 in region-wise F1 score (Tables 4, 5).

**TABLE 4:** Comparison of dice accuracy for different CSVDs.

	Lacune	White matter hyperintensity	Infarction	Cerebral microbleed
Doctor A	0.298	0.614	0.717	0.549
Doctor B	0.578	0.670	0.747	0.715
Doctor C	0.506	0.579	0.758	0.613
Doctor D	0.354	0.521	0.754	0.672
Doctor E	0.412	0.596	0.690	0.514
Doctor F	0.388	0.509	0.615	0.456
Average	0.423	0.582	0.714	0.586
Our model	0.496	0.666	0.728	0.503

*Comparison of dice accuracy for different CSVDs of 30 patients according to the predictions made by six doctors (Neuroradiologists) and by our model, concerning the reference standard.*

**TABLE 5:** Comparison of region-wise F1 score for different CSVDs.

	Lacune	White matter hyperintensity	Infarction	Cerebral microbleed
Doctor A	0.375	0.660	0.817	0.785
Doctor B	0.676	0.722	0.905	0.897
Doctor C	0.633	0.661	0.905	0.809
Doctor D	0.41	0.668	0.921	0.797
Doctor E	0.518	0.603	0.836	0.662
Doctor F	0.536	0.518	0.652	0.623
Average	0.525	0.639	0.839	0.762
Our model	0.683	0.644	0.859	0.713

*Comparison of region-wise F1 score for different CSVDs of 30 patients according to the predictions made by six doctors (Neuroradiologists) and by our model, with respect to reference standard. Each lesion is treated equally regardless of size, and the prediction is classified as a true positive when one or more pixel overlaps with the reference standard.*

For each patient, our DLS system probably can process the images and output a volumetric prediction on the location of four CSVD diseases (lacune, WMH, subcortical infarction and cerebral microbleed) within a mean duration of 4.4 seconds (Table 6). The mean time used by each of the six doctors to draw masks of a single patient to produce a volumetric prediction ranges from 330 s to over 1,000 s. Compared to the segmentation independently made by six doctors, the predictions made by our model are over a hundred times faster and attained a higher dice accuracy and region-wise F1 score on average. Our DLS can suggest the diagnosis and draw the segmentation masks for over a hundred patients in the average time used by a doctor to do the same for one patient.

**TABLE 6:** Credentials of doctors and time spent on the segmentation of 30 patients.

	Experience	Job title	Average time spent patient (in seconds, *n* = 30)
Doctor A	3 years	Resident Physician	1094/case
Doctor B	9 years	Attending Physician	662/case
Doctor C	18 years	Chief Physician	594/case
Doctor D	14 years	Chief Physician	418/case
Doctor E	3 years	Resident Physician	718/case
Doctor F	3 years	Resident Physician	330/case
Average	8 years		636/case
Our model			4.4/case

*Credentials of doctors (Neuroradiologists) and time spent on the segmentation of 30 patients, including the drawing of segmentation masks, compared with the time required by our model to perform image processing and prediction.*

## Discussion

In this paper, by using T1-weighted, T2^∗^, T2-FLAIR, and DWI b1000 images, we trained a DLS to draw the presented diseases area of lacune, WMH, subcortical infarction, and cerebral microbleed. We compare its performance with that of six doctors, using the reference standard set unanimously by three senior doctors. The results are evaluated based on the classical dice score, a modified patch-wise dice score, which allows for minor uncertainties in the neighborhood of a few pixels, as well as the region-wise F1 score, which may be a more suitable indication of success in the detection of lesions. The results show that our model can diagnose and draw the segmentation masks of multiple CSVDs more reliably, and over a hundred times faster than doctors with an average experience of eight years.

This indicates that if patients trust the segmentation set by a panel of three senior doctors, they have reason to prefer the advice of our model over the opinion of an average doctor with few years of experience. It is also worthy to note that all six doctors are from Beijing Tiantan Hospital, which is a leading hospital in China and hosts one of the most extensive neurosurgical bases in China. Hence, these doctors are likely to be more rigorously trained than doctors from an average hospital in less affluent parts of China.

The results of our comparison support to the case that appropriately trained DLS can be trusted to the same extent as one would trust a doctor with a few years of experience, regardless of whether the emphasis is placed on the segmentation or detection of lesions. However, we want to emphasize that the proposed DLS is not aimed to replace doctors but meant to serve as a guide to doctors, where inconspicuous anomalies detected by the computer will warrant a closer look.

### Limitation

In what follows, we discuss the limitations of our work and recommend possible improvements. First, as the testing dataset gets larger, the DLS is likely to have superior performance. However, it should be noted that, while all annotations made in the dataset have been endorsed by an associate chief physician with at least 10 years of experience, they are initially prepared by junior doctors with relatively less experience. Given that our model has been trained on these data, it is more likely to make predictions similar to these doctors rather than the senior doctors prescribing the reference standard. Had the model been trained on a vast number of images annotated by those senior doctors, its segmentation will likely bear a much closer resemblance to theirs.

Second, our model is compared against the performance of six doctors from a single country, and their average performance may not be representative of the average performance of all doctors globally. More doctors from a variety of hospitals and across different countries can be sought to participate in the clinical evaluation. Additionally, more patients can also be added to the evaluation dataset, so the results of our model, as well as the doctors, can be analyzed with greater certainty.

Third, our model is trained primarily to diagnose only four types of small vessel diseases. Therefore, our results cannot be generalized to compare the reliability of a DLS relative to the overall proficiency of a practicing doctor. Moreover, we do not deny the fact that our model is unable to propose a treatment, unlike a human doctor. Our study can be extended to train models capable of predicting a wide variety of medical anomalies. Besides, artificial intelligence is now progressing toward treatment planning and may be able to recommend solutions to their diagnosis in the future.

We reiterate that the purpose of this study is not to assert that DLS is more reliable than doctors. Instead, it is to propose that an adequately trained deep learning model can supplement the diagnosis of an attending doctor, and that one may heed its advice in the same way as one would respond to the words of a trained doctor.

## Conclusion and Contributions

This study is a preliminary study focusing on lesion segmentation and identification. Previous studies showed the individual feature of CSVD is associated with incident ischemic and hemorrhagic stroke, dementia, and depression. Combinations of two features were more strongly associated with stroke than any specific feature (Pantoni, 2010; Go et al., 2012). So our model covered different types of lesions. According to the current results, the model can obtain lesion recognition at the level of attending physicians, which can significantly reduce the repetitive labor of physicians. For the further clinical application, with the help of this system, it may help clinical doctor fast categorizing and masking cerebral small vessel disease less time consuming, laborious, and subjective. Based on our DLS model, not only the location of the disease can be determined by the segmentation mask, but also the volume of lesions, which is critical in dosage prescription or clinical decision support systems (Belard et al., 2017).

## Data Availability Statement

The raw data supporting the conclusions of this article will be made available by the authors, without undue reservation, to any qualified researcher.

## Ethics Statement

The studies involving human participants were reviewed and approved by Beijing Tiantan Ethics Committee. Written informed consent for participation was not required for this study in accordance with the national legislation and the institutional requirements. Written informed consent was not obtained from the individual(s) for the publication of any potentially identifiable images or data included in this article.

## Author Contributions

YD and WS wrote the initial draft of the manuscript. WS provided both figures and made preliminary revision. LL (Main contributor), PL, JJ, and ZW make contribution in the DLS development and medical test organization. LL, QW, ZW, PL, and JJ made preliminary revision. YL, KH, and YW made crucial revision. All authors together planned the manuscript, critically revised the initial draft, and made final improvements prior to submission.

## Conflict of Interest

The authors declare that the research was conducted in the absence of any commercial or financial relationships that could be construed as a potential conflict of interest.
