# Supplementary material for: Primary Categorizing and Masking Cerebral Small Vessel Disease Based on “Deep Learning System”
Source: Front Neuroinform. 2020 May 25;14:17. doi: 10.3389/fninf.2020.00017 (PMC7261942; doi:10.3389/fninf.2020.00017)
Supplement: Supplementary file 1 [file Data_Sheet_1.docx]

Table S1: Data distribution and sequence parameters from multiple clinical centres.

|  | #Patients | | | |  | In-plane resolution (mm*mm/pixel) | | |  |
| --- | --- | --- | --- | --- | --- | --- | --- | --- | --- |
| Center code | infarction | lacune | WMH | microbleed | Thickness (mm) | T1WI, T2WI, and FLAIR | DWI | T2* | Scanner type |
| 01 | 73 | 60 | 60 | 0 | 6, 6.5 | 0.47*0.47 | 0.94*0.94 | / | GE Discovery MR750-3.0T |
| 02 | 90 | 77 | 77 | 37 | 8 | 0.43*0.43 | 0.83*0.83; 0.90*0.90 | 0.43*0.43 | Philips Achieva-1.5T |
| 03 | 94 | 94 | 94 | 0 | 6, 6.5, 7.5 | 0.50*0.50 | 0.55*0.55 | / | Philips Achieva-1.5T |
| 04 | 72 | 72 | 72 | 29 | 6.5 | 0.50*0.50 | 0.94*0.94 | 0.45*0.45 | Philips Achieva-1.5T |
| 05 | 48 | 48 | 48 | 33 | 6.5, 8 | 0.56*0.56 | 0.94*0.94 | 0.47*0.47 | GE Optima MR360-1.5T |
| 06 | 112 | 106 | 106 | 44 | 6.5 | 0.47*0.47 | 0.94*0.94 | 0.47*0.47 | GE Optima MR360-3.0T |
| 07 | 95 | 43 | 43 | 43 | 6, 6.5, 7.5 | 0.43*0.43 | 1.20*1.20 | 0.43*0.43 | Siemens Verio-3.0T |
| 08 | 52 | 52 | 52 | 30 | 6.0, 6.5 | 0.90*0.90 | 0.9*0.9 | 0.90*0.90 | Philips Achieva-1.5T |
| 09 | 56 | 56 | 56 | 0 | 6.5, 7.0 | 0.72*0.72 | 1.20*1.20 | / | Siemens Avanto-1.5T |
| 10 | 79 | 39 | 39 | 27 | 7 | 0.56*0.56 | 0.94*0.94 | 0.47*0.47 | GE Discovery MR750-3.0T |
| 11 | 64 | 41 | 41 | 47 | 6 | 0.45*0.45 | 1.20*1.20 | 0.45*0.45 | Siemens Avanto-1.5T |
| 12 | 104 | 104 | 104 | 0 | 6.5, 7.8, 8.0 | 0.47*0.47 | 0.90*0.90 | / | Philips-Achieva-1.5 T |
| 13 | 71 | 33 | 33 | 69 | 6 | (0.72*0.72) | 1.20*1.20 | 0.90*0.90 | Siemens TrioTim-3.0T |
